# A Numerical Study of the Mechanical Behavior of Jointed Soft Rocks under Triaxial Loading Using a Bonded Particle Model

**DOI:** 10.3390/ma17194842

**Published:** 2024-09-30

**Authors:** Mingxing Liu, Yijian Xu, Xiaohu Gao, Jie Fu, Xingyan Liu, Enlong Liu

**Affiliations:** 1 School of Civil Engineering and Architecture, East China Jiaotong University, Nanchang 330013, China; 2 Jiangxi Key Laboratory of Disaster Prevention-Mitigation and Emergency Management, East China Jiaotong University, Nanchang 330013, China; 3 College of Water Conservancy & Architectural Engineering, Shihezi University, Shihezi 832003, China; 4 College of Water Resource and Hydropower, Sichuan University, Chengdu 610065, China

**Keywords:** bonded particle model, jointed soft rock, mesoscopic property, triaxial loading

## Abstract

In order to master the strength and deformation characteristics, including the macro–micro failure mechanism of soft rock samples with penetrating joints under triaxial loading, a series of numerical triaxial tests have been carried out. The strength and deformation characteristics, failure modes, crack propagation, distribution of force chains, and the influences of joint dip angles and confining pressures have been analyzed and compared with the laboratory test results. The results show that (1) the residual strength ratio of jointed rock samples generally increases first and then decreases with the increase in joint dip angles under the same confining pressure and reaches the maximum value around 23–24°. Poisson’s ratio increases with the increase in the confining pressure or the joint dip angle. The elastic modulus increases with the increase in the confining pressure and decreases with the increase in the joint dip angle. (2) The jointed rock samples with different joint dip angles compact with relatively small volumetric strains and then dilate up to failure with relatively large volume expansions. Lower confining pressure and smaller dip angles will lead to a more pronounced dilation phenomenon and less obvious volume shrinkage rules. (3) The low-angle jointed rock samples all exhibit the X-type shear failure. The jointed rock samples with a joint dip angle of 45° exhibit hybrid failure with both slippage and shearing, which are controlled by both the matrix and the joint. (4) The change in the number of cracks includes three stages: the slow crack initiation stage, rapid growth stage, and crack coalescence stage. The total number of shear or tensile cracks all decrease with an increase in the joint dip angles, with the number of tensile cracks being approximately twice that of shear cracks. The tension cracks are mostly horizontal, and the shear cracks are mostly vertical. (5) The number of force chains shows a decreasing trend after the cracks begin to grow. The jointed rock samples for the intact, 15° and 30° cases all form a main force chain during the failure process, while there is no main force chain for the 45° case.

## 1. Introduction

Rocks in engineering are often characterized by numerous cracks, which result from geological processes, external loads, weathering, and other factors. As these cracks continue to develop, some rocks exhibit penetrated joints, which significantly impact their mechanical properties and often play a decisive role in the stability and safety of rock mass engineering projects. Therefore, studying the deformation and failure mechanisms of jointed rocks with penetrated joints is of great practical importance. Experimental studies are a common approach to investigating the mechanical properties of jointed rocks. While the data and results obtained from experiments are generally reliable, it is challenging to observe and analyze the internal characteristics and mechanisms. Additionally, the properties of rocks, particularly the joints, can exhibit considerable variability, leading to potential errors in the experimental results. In contrast, with numerical simulation methods, the difficulties of sampling and strength discreteness can be overcome, and the mesoscopic mechanism can be studied more easily. Most numerical methods, such as the finite element method, cannot describe well the discontinuous deformation of jointed rocks [1,2], while the discrete element method based on the bonded particle model [3] can provide an effective simulation of rock mechanical properties, particularly from a mesoscopic perspective [4,5,6,7]. This approach can enhance our understanding of the macro–micro deformation and failure mechanisms of jointed rock masses.

Based on the discontinuities in rock masses, Cundall [8] introduced the discrete element method (DEM) in 1971. The DEM constructs a model composed of interacting units segmented by joint fractures. Farahmand et al. [9] proposed a discrete element model that considers particle scale and material properties. Damjanac et al. [10] introduced the Synthetic Rock Mass (SRM) concept for simulating natural joints. Chiu et al. [11] developed the Particle Interface Model (PIM), which does not require inverse analysis of parameter values. Subsequently, the discrete element method evolved into a numerical analysis software known as the Particle Flow Code (PFC) [3]. Some researchers have partially studied the static mechanical properties of various jointed rocks based on the bonded particle model. Liu et al. [12] simulated the failure mechanisms of cemented sandstone during the shearing process using PFC^3D^ considering the parallel bond model. By varying the cemented radius, parallel bond stiffness, and cemented volume of the particles, the significance of cemented properties on the bearing capacity of the sandstone structures was highlighted. Other researchers [13,14,15] incorporated different angles of joint surfaces into the particle model to investigate the effects of the geometric parameters of joints—including direction, density, size distribution, number, etc.—on the uniaxial compression strength of samples. They identified four failure modes for jointed rocks under uniaxial compression: complete tensile failure, sliding failure along the joint plane, deformation failure along the vertical joint plane, and mixed tension-slip failure. Xia et al. [16] analyzed the damage process of rough joints under different contact states through numerical direct shear tests, observing a significant increase in the number of microcracks once the peak shear stress was reached. Fan et al. [17] examined the effects of flaws on the cracking behavior of rocks under uniaxial compression, utilizing bonded particle models. They found that flaws significantly influence the peak stress, the crack initiation stress, and the number of microcracks, while the local stress distribution effectively explains the cracking behavior. From a microscopic perspective, Li and Sun et al. [18,19] investigated the crack propagation patterns and energy conversion characteristics of rock specimens under triaxial loading via the discrete element method. Their findings revealed that energy transformation occurs simultaneously with the initiation and propagation of cracks, resulting in an increase in the numbers of both tangential and normal cracks as dissipated energy increases. Meanwhile, Xu and Zhou et al. [20,21] conducted uniaxial compression simulations on rock samples containing single fractures to determine an optimal calibration method for micro parameters in the PFC. They established a connection between the micro parameters of the bonded particle model and the macroscopic physical and mechanical properties of materials.

The aforementioned researchers have conducted numerous numerical studies on the static properties of jointed rock, focusing on aspects such as joint plane geometry, crack propagation, particle cementation, and failure modes. They have achieved significant results in these areas. However, the deformation and failure mechanism of through-type jointed rock are still not well understood, and research on the mesoscopic mechanism remains incomplete. Furthermore, there is a limited amount of research on jointed soft rock. Therefore, based on indoor triaxial tests on jointed rock samples, a series of numerical static triaxial tests were conducted using the discrete element method based on the bonded particle model. These tests aimed to investigate the strength and deformation characteristics, as well as the macro–micro failure mechanisms, of soft rock influenced by through-type joints with various dip angles.

## 2. Numerical Test Scheme

This study is based on static triaxial tests conducted on artificially prepared cylindrical samples of soft rock with a single penetrated joint [22,23]. The samples have a density of 2100 kg/m^3^, a matrix elastic modulus of approximately 125 MPa, and a Poisson’s ratio of about 0.35. The testing was performed using the GCTS stress path vibration triaxial instrument. Initially, static triaxial compression tests were carried out on both intact and jointed soft rock samples with joint dip angles of 15°, 30°, and 45°. The static deformation and failure mechanisms of jointed soft rock were then analyzed from a macroscopic phenomenological perspective.

**Figure 1:**
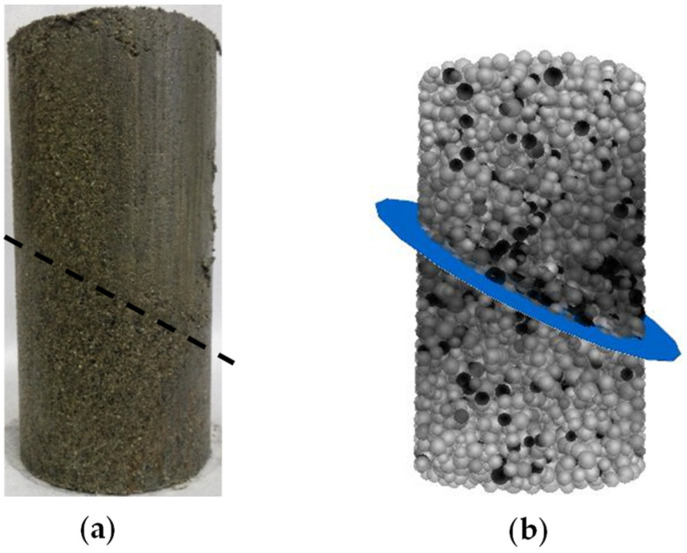
Jointed rock sample (with a joint dip angle of 30°) for laboratory tests and bonded particle model. (**a**) Laboratory testing sample; (**b**) bonded particle model.

PFC^3D^, a software based on the discrete element method, was used to model the mechanical behavior of granular materials, such as geotechnical materials, through interactions between particles. In this model, particles are treated as rigid spheres, and interactions are governed by Newton’s second law and the contact constitutive relation between particles. Based on the results of the static test, a numerical model was established using PFC^3D^. Firstly, a cylindrical calculation boundary of ϕ50 mm×100 mm was created. Then, spherical particles with radii ranging from 1.5 mm to 2.5 mm, distributed according to a normal distribution, were generated within the calculation volume. The particle density was set to 2300 kg/m^3^, and a damping coefficient of 0.5 was adopted. Bond models between particles include the contact bond model and the parallel bond model, and the parallel bond model was chosen based on previous studies using the discrete element method for rocks [24,25,26]. The joint plane was generated using methods such as particle removal, cementing material generation, and the smooth joint model. Considering the actual joint plane, the smooth parallel joint model was selected. All joint planes intersect the center of the cylindrical specimen. As a result, a numerical model of jointed soft rock consisting of 5055 particles was generated. Figure 1b illustrates the particle model of the jointed rock sample with a joint dip angle of 30°, where the blue plane indicates the joint plane (Figure 1a shows an indoor test sample, where a black dotted line represents the joint plane). Lateral confining pressure was applied directly through servo control, while axial load was applied by adding the loading walls to both the upper and lower surfaces of the model and controlling axial displacement. The numerical calculations were terminated when the axial strain exceeded 10%.

**Figure 2:**
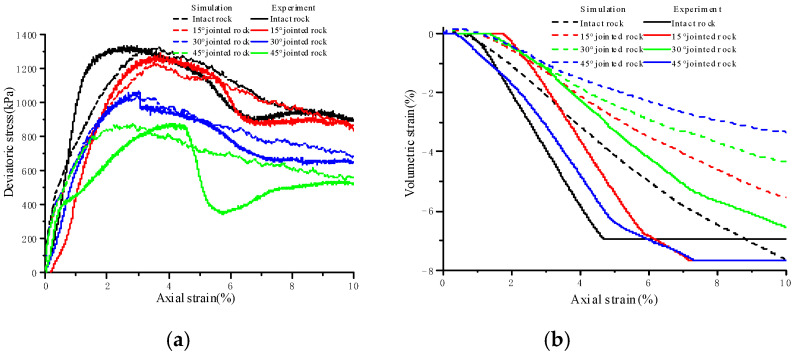
Comparison of simulated and experimental stress–strain curves under a confining pressure of 100 kPa. (**a**) Deviatoric stress–axial strain curves; (**b**) volumetric strain–axial strain curves.

The meso-parameters of the particle model influence the macro-meso mechanical properties of jointed soft rocks. Initially, a benchmark set of meso-parameters was established based on the results of laboratory triaxial tests and the calibration results of PFC^3D^ meso-parameters for sandstone samples in previous studies [27,28]. Then, these parameters were refined through sensitivity analysis of the influence rules of particle meso-parameters on mechanical parameters such as elastic modulus, Poisson’s ratio, peak strength, and residual strength of jointed soft rocks. The contact stiffness between particles was adjusted to align with the elastic modulus observed in laboratory samples, while the stiffness ratio *k*_n_/*k*_s_ between particles was fine tuned to match Poisson’s ratio. The final parameters for the jointed soft rock sample particle model are detailed in Table 1. Figure 2 compares the simulated stress–strain curves with experimental data. It was found that the simulated peak strength, residual strength, strain softening behavior, and volumetric strain characteristics are consistent with the laboratory test results. This suggests a reliable representation of the mechanical properties of jointed soft rock samples.

In this study, static triaxial compression numerical tests were performed on intact and jointed soft rock samples with joint dip angles of 15°, 30°, and 45° under confining pressures of 100 kPa, 200 kPa, 300 kPa, and 400 kPa. The obtained results were compared with laboratory test results. Then, a comprehensive analysis was conducted to examine stress–strain characteristics, failure modes, crack patterns, and force chain behaviors. These findings will help reveal the macro–micro mechanical properties of the jointed soft rock samples.

## 3. Strength and Deformation Characteristics

Figure 2 presents a comparison between the simulated and experimental stress–strain curves and the volumetric strain–axial strain curves. Axial stress is calculated based on the contact force exerted by the loading wall and the cross-sectional area of the samples, while radial strain is derived from the relative change in the radii of particles on the outermost surface, and axial strain is obtained from the relative change in sample height. It can be observed that the simulated stress–strain curves effectively capture the primary strength and deformation characteristics of jointed soft rock, although there is a discrepancy in the magnitude of elastic modulus. The simulation results demonstrate strain-softening behavior and a certain level of ductility in jointed soft rock. Under identical confining pressures, the elastic modulus, peak strength, residual strength, and volumetric expansion decrease as joint dip angles increase. Jointed soft rock samples with varying inclinations initially exhibit minor volumetric contraction, followed by continuous volumetric expansion until failure. Notably, for the sample inclined at 45°, significant slip along the joint plane results in a measured volumetric expansion that is larger than the actual value.

### 3.1. Strength Characteristics

**Figure 3:**
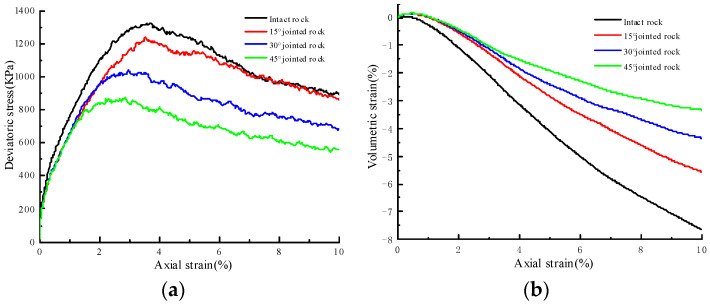
Simulated stress–strain curves of jointed rock samples under the confining pressure of 100 kPa. (**a**) Deviatoric stress–axial strain curves; (**b**) volumetric strain–axial strain curves.

**Figure 4:**
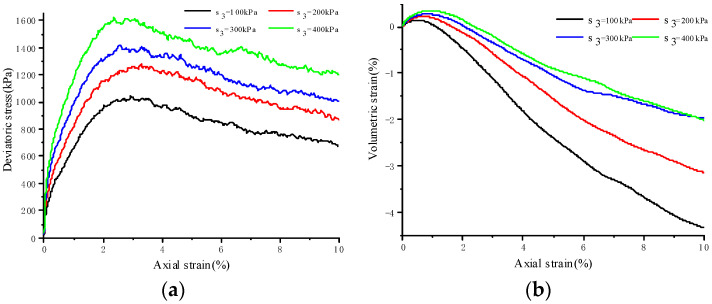
Simulated stress–strain curves of 30° jointed rock samples under different confining pressures. (**a**) Deviatoric stress–axial strain curves; (**b**) volumetric strain–axial strain curves.

Figure 3 illustrates the stress–strain behavior of jointed soft rock at various dip angles and under a confining pressure of 100 kPa, while Figure 4 depicts the stress–strain response of jointed soft rock at a dip angle of 30° and under different confining pressures. Residual strengths are determined as the deviatoric stress when the axial strain reaches 10%. It is observed that both peak and residual strengths increase with higher confining pressure for a given joint dip angle but decrease with increasing joint dip angles under the same confining pressure. The stress–strain curves under different conditions all exhibit strain-softening characteristics, followed by a nearly horizontal plastic flow stage in the post-peak phase. These observations are consistent with the binary medium theory, which posits that bonding elements in the rock sample gradually transform into frictional elements during loading, leading to strain-softening behavior.

**Figure 5:**
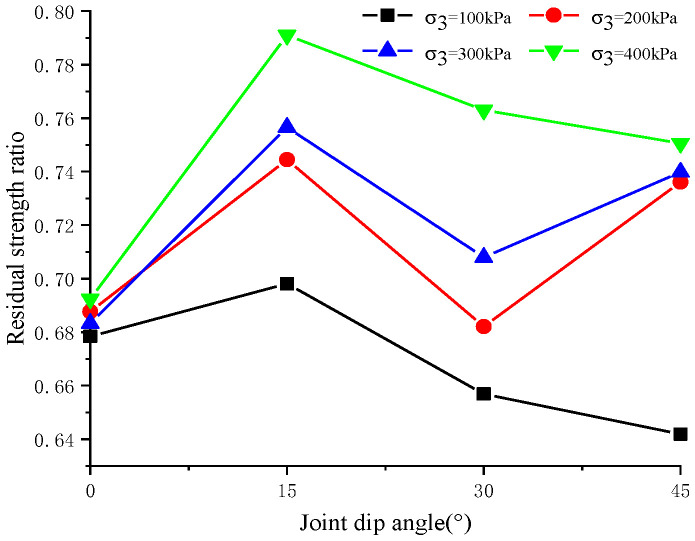
Residual strength ratio of jointed rock samples with different joint angles and different confining pressures.

In Figure 3a, it is evident that at a constant confining pressure, the increase in peak strength and residual strength of rock samples exhibits a non-linear relationship with the decrease in joint angle. Figure 4a shows that for the same joint inclination, strength increases non-linearly with rising confining pressure, with the increase in residual strength being more pronounced than the increase in peak strength. The residual strength ratio, defined as the ratio of residual strength to peak strength, is determined for jointed rock samples under various joint angles and confining pressures (see Figure 5). It is observed that this ratio initially increases and then decreases as the joint angle increases at a constant confining pressure, reaching its maximum value between 0° and 30°. To precisely identify the range of the maximum residual strength ratio, static loading simulations are conducted under a confining pressure of 100 kPa with systematic variation of the joint inclination angle. The analysis indicates that the maximum residual strength ratio occurs at approximately 24.1°. Additionally, with changes in confining pressure, the maximum residual strength ratio for jointed rock samples is found to be between 23° and 24°, as detailed in Table 2.

### 3.2. Deformation Characteristics

It can be observed in Figure 3b and Figure 4b that volume shrinkage occurs during the initial loading stage of jointed rock samples, specifically when the rock samples are undergoing compaction. At equivalent confining pressures, the maximum volume shrinkage is largest in rock samples with a 45° joint angle, while the minimum is seen in intact rock samples. Although joint surfaces influence the volume shrinkage of rock samples to some extent, there is no significant difference in volume shrinkage between samples with joint angles ranging from 15° to 45°. Following the volume shrinkage phase, the rock samples undergo a period of expansion leading to eventual failure. This dilatancy phenomenon [29,30] exhibits a significantly greater magnitude compared to the preceding shrinkage phase. Under identical confining pressures, the volume expansion at failure demonstrates an approximately linear decrease with increasing joint dip angle. This observation aligns with the data presented in Table 3, which indicates a reduction in crack occurrence as the joint inclination increases. This trend can be attributed to a gradual decrease in joint strength with an increasing joint dip angle, coupled with increased slip along the joint surfaces within the rock matrix and reduced deformation within the matrix.

Figure 4b illustrates that, for the same joint inclination, both the maximum volumetric shrinkage and the corresponding axial strain of jointed rock samples increase with confining pressure, indicating a higher degree of compaction at higher confining pressures. Conversely, volumetric expansion at failure decreases with increasing confining pressure under the same joint inclination; namely, rock samples subjected to high confining pressure experience less volumetric expansion for the same axial strain. 

Poisson’s ratio and the elastic modulus are critical elastic parameters of jointed rock samples. Based on the calibration method for meso-parameters proposed by Sun et al. [31] combined with binary medium theory and plane stress hypothesis, the formulas for calculating Poisson’s ratio and the elastic modulus are derived by considering axial strain before 0.25% as elastic deformation. The formulas are as follows:

(1) $$E=(1-\upsilon^{2})\frac{\Delta \sigma_{y}}{\Delta \varepsilon_{y}}$$

(2) $$\upsilon =\frac{\Delta \varepsilon_{r}}{\Delta \varepsilon_{y}+\Delta \varepsilon_{r}}$$

Herein, E is the macroscopic elastic modulus of the jointed rock sample; υ is the macroscopic Poisson’s ratio; $\Delta \varepsilon_{r}$ and $\Delta \varepsilon_{y}$ are the increments of the radial strain and axial strain, respectively; and $\Delta \sigma_{y}$ represents the increment in deviatoric stress.

**Figure 6:**
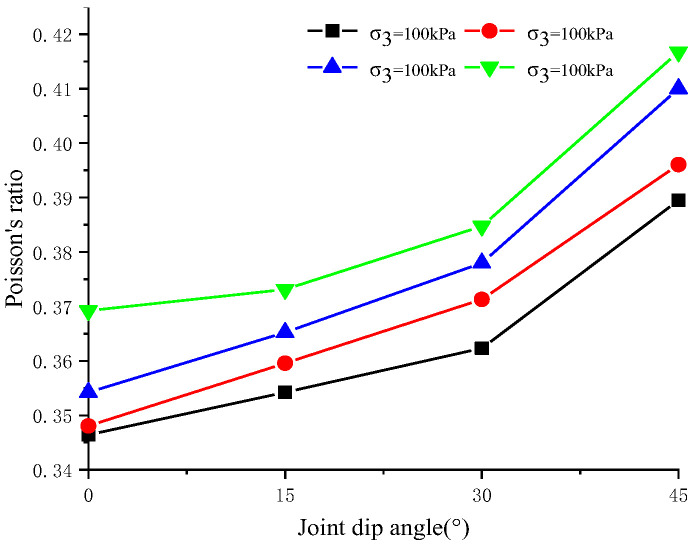
Poisson’s ratio at different joint dip angles and under different confining pressures.

Figure 6 illustrates the influence of different confining pressures and joint dip angles on Poisson’s ratio of jointed soft rocks. It is observed that for a given joint dip angle, Poisson’s ratio increases with rising confining pressure. Similarly, for a constant confining pressure, an increase in joint dip angle results in an increase in Poisson’s ratio. These findings indicate that both confining pressure and joint conditions impact Poisson’s ratio of rock samples; however, it can be concluded that the effect of the joint angle is more significant than that of confining pressure on this parameter.

**Figure 7:**
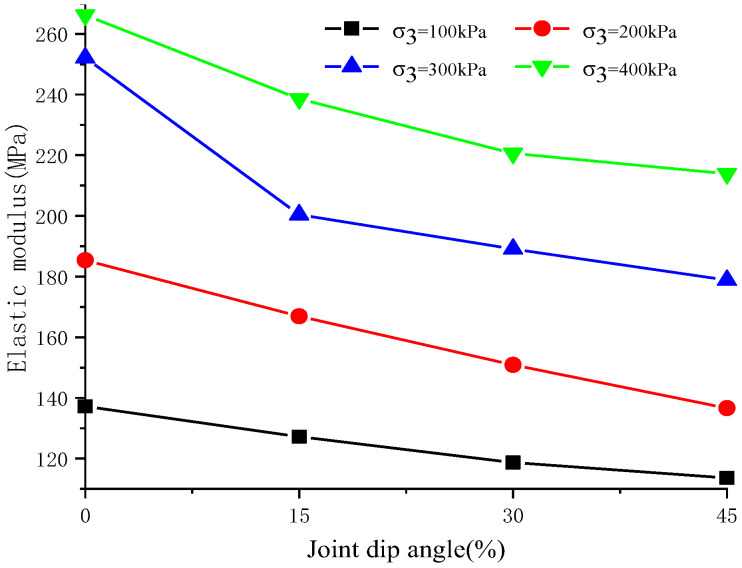
Elastic modulus at different joint dip angles under different confining pressures.

Figure 7 presents the elastic modulus results obtained from tests conducted on soft rocks with varying joint dip angles and different confining pressures. It is evident that at a fixed joint dip angle, the elastic modulus increases with confining pressure, indicating that jointed rock samples exhibit greater resistance to deformation under high confining pressures. Conversely, with constant confining pressure, an increase in joint dip angle leads to a decrease in elastic modulus values, with 45° jointed samples showing the lowest elastic modulus among all the tested configurations. In summary, both joint dip angles and confining pressures influence the elastic modulus behavior of jointed rock samples; nevertheless, the effect induced by variations in confining pressure outweighs that caused by changes in joint dip angles.

## 4. Failure Mode, Crack Propagation, and Force Chain Distribution

### 4.1. Failure Mode Analysis of Jointed Soft Rock

**Figure 8:**
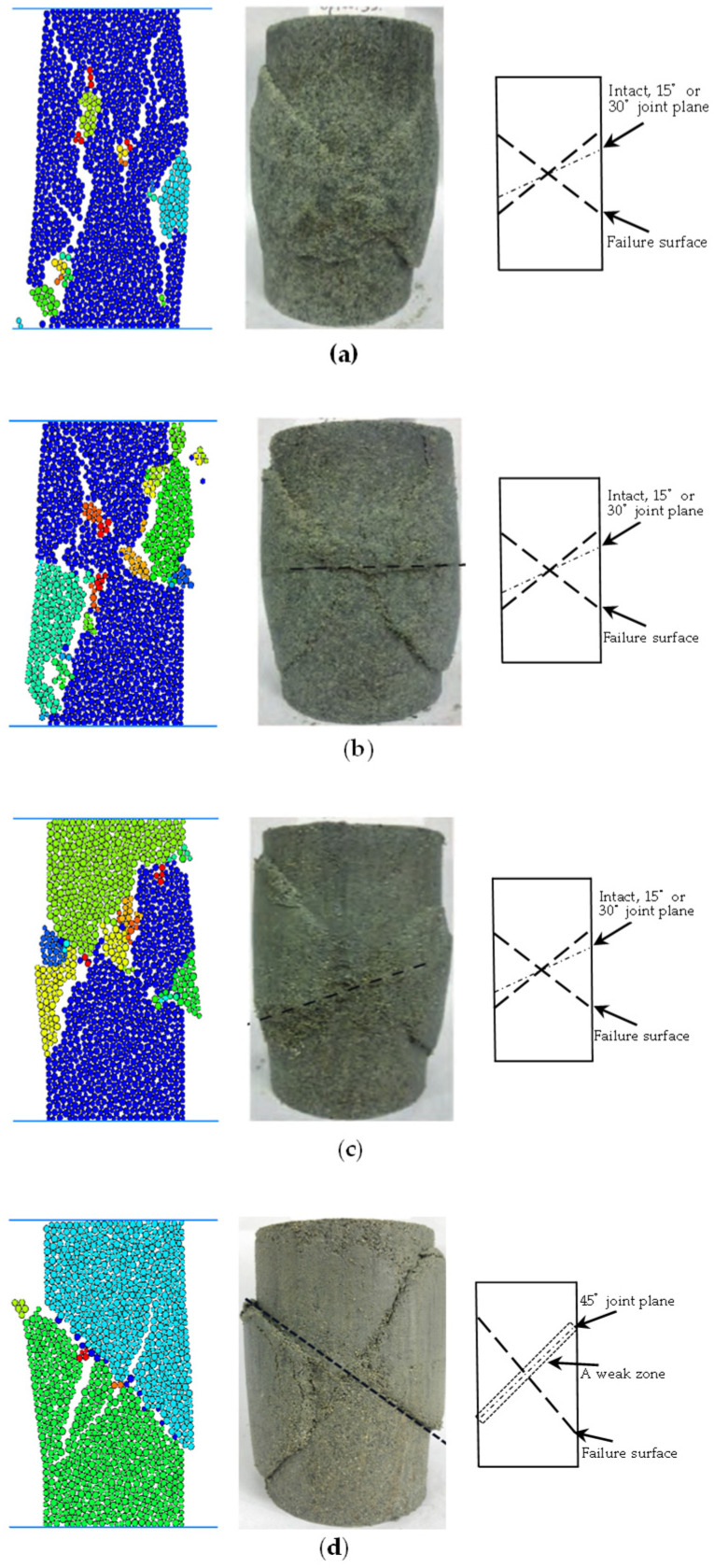
The simulated and experimental failure modes under the confining pressure of 100 kPa and the failure mechanism diagram. (**a**) Intact rock sample, (**b**) at a joint dip angle of 15°, (**c**) a joint dip angle of 30°, (**d**) and a joint dip angle of 45°.

Figure 8 illustrates the simulated and experimental failure modes and failure mechanisms of soft rock with varying joint inclinations under a confining pressure of 100 kPa. When particle bonds are damaged, cracks develop due to shear or tension forces along these bonds. The total number of cracks and the cracks’ orientations can be monitored. Under identical confining pressure, rock samples with intact structures and 15° and 30° joint inclinations all exhibit “X”-type shear failures within the matrix. Conversely, rock samples with a 45° joint inclination initially show significant slip deformation along the joint plane, followed by the formation of a shear band within the matrix, oriented opposite to the joint inclination. The simulation results reveal that besides the primary shear band evident in laboratory tests, secondary shear cracks also contribute significantly to the mechanical properties of jointed soft rock. For lower joint inclinations (e.g., 15° and 30°), the larger angle between the direction of the joint plane and the loading direction results in reduced shear stress on the joint plane, thereby enhancing its resistance to deformation and failure. In these cases, the jointed rock sample does not exhibit noticeable slips along the joint plane, and an “X”-type shear crack directly traverses the structural plane. The strength of these samples is predominantly influenced by the matrix, with strain softening mainly attributed to damage and softening of the matrix. Thus, the failure mode resembles that of intact rock samples, characterized by “X”-type shear failure. At a 45° joint inclination, the angle between the joint plane and the loading direction is larger. In this case, stress on the joint plane exceeds bond strength between structural surfaces, while the stress on cement bonds among particles in the matrix falls far short of its strength, causing a large slip along the joint plane. The slip deformation gradually forms a weak zone along the joint plane. Only when the strength of the joint plane in the slip direction approaches that of the matrix does a shear zone opposite to the joint plane emerge within the matrix. However, due to the lower shear strength of the joint plane in the opposite direction, the bearing capacity of the 45° jointed rock sample is limited. Consequently, the strength and failure of this sample are primarily governed by both the joint plane and matrix, with a predominant influence from the joint plane. Moreover, strain softening is primarily driven by damage and softening within the matrix. 

### 4.2. Distribution Pattern of Cracks in Jointed Soft Rock

**Figure 9:**
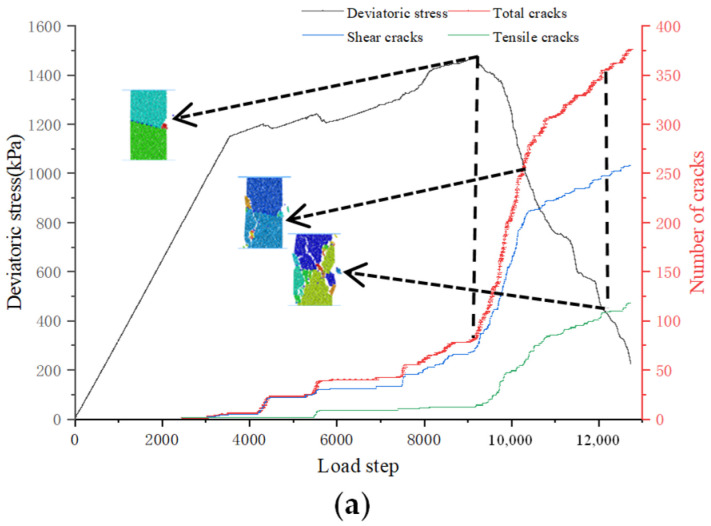

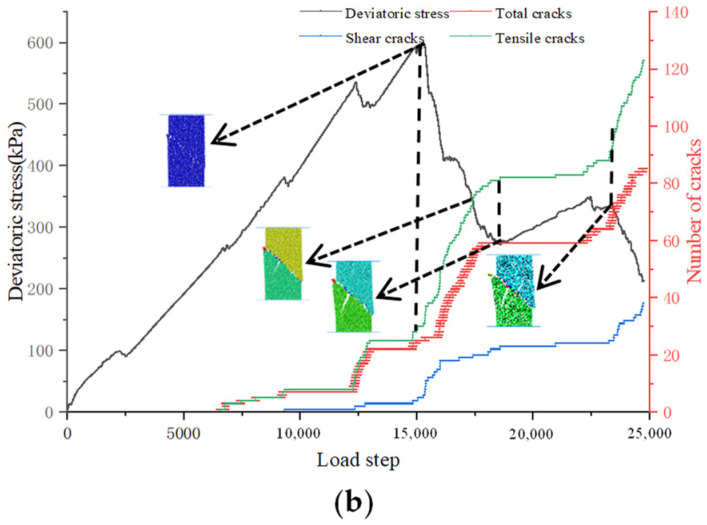
Evolution curve of number of cracks in 15° and 45° rock samples under 100 kPa confining pressure. (**a**) at a joint dip angle of 15° (**b**) and a joint dip angle of 45°.

**Figure 10:**
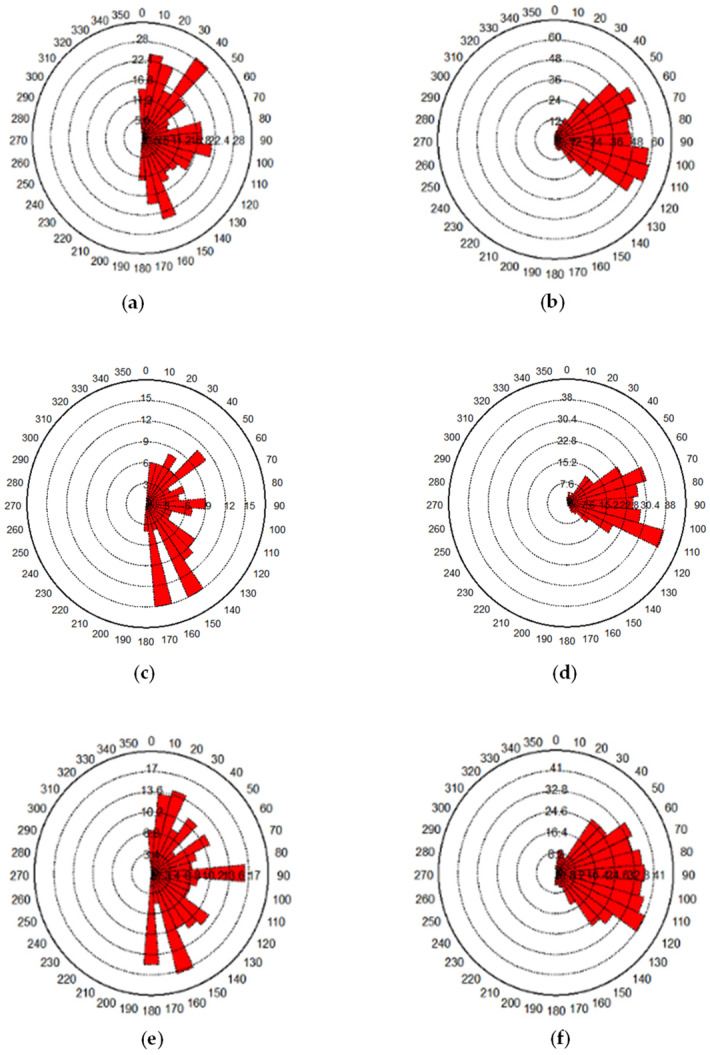

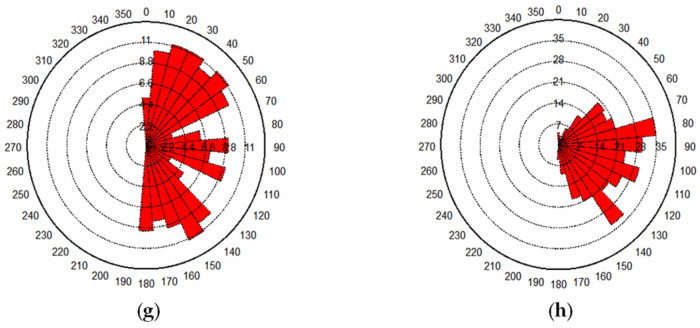
Rose diagram of crack orientation for rock samples under a confining pressure of 100 kPa. (**a**) Tensile cracks of intact rocks; (**b**) shear cracks of intact rocks; (**c**) tensile cracks of 15° jointed rocks; (**d**) shear cracks of 15° jointed rocks; (**e**) tensile cracks of 30° jointed rocks; (**f**) shear cracks of 30° jointed rocks; (**g**) tensile cracks of 45° jointed rocks; (**h**) shear cracks of 45° jointed rocks.

During the loading process of jointed soft rock, the initiation of cracks indicates the rupture of cementation bonds between particles. In the initial loading stage, the rock sample predominantly experiences axial compression and slight radial displacement within the matrix. As loading continues, internal damage within the rock sample escalates, leading to the disruption of force chains and the fracturing of cementation bonds between particles. Figure 9 illustrates the variation in the number of cracks for rock samples with inclinations of 15° and 45° under a confining pressure of 100 kPa. It is evident that the crack propagation in jointed rock samples with different inclinations exhibits similar characteristics, consisting of three distinct stages. The first stage is characterized by slow crack growth. During this stage, from the beginning of crack initiation to the peak stress, the number of cracks increases slowly and the internal matrix structure within the rock sample maintains substantial integrity. The second stage involves rapid crack propagation, extending from the peak stress to the onset of shear zone formation. In this stage, the number of cracks increases rapidly, and shear zones are about to form. The third stage is marked by crack penetration and failure, during which cracks progressively merge to form a shear failure zone. Although the number of cracks still increases rapidly in this stage, the rate of growth is slower compared to the second stage. Table 3 presents the number of cracks in jointed soft rock samples with various dip angles under a confining pressure of 100 kPa. It shows a decrease in the total number of cracks as the joint dip angle increases under the same confining pressure, as well as in the total number of shear or tensile cracks. This trend is consistent with the observation in Figure 7, which shows that the degree of breakage in samples decreases with increasing joint dip angles. Furthermore, each jointed rock sample exhibits approximately twice as many shear cracks as tensile cracks, indicating that mesoscopic failure is predominantly attributed to shearing. Figure 10 displays a rose diagram of crack orientation for jointed rock samples with different dip angles under the confining pressure of 100 kPa. It reveals that no matter whether the joint dip angle is large or small, shear cracks predominantly occur at angles between 70° and 110°, mainly near the axial loading direction. Conversely, tensile cracks are primarily observed at angles between 0° and 20°, and 160° and 180°, closer to the horizontal direction.

### 4.3. Evolution of Force Chains in Jointed Soft Rock

**Figure 11:**
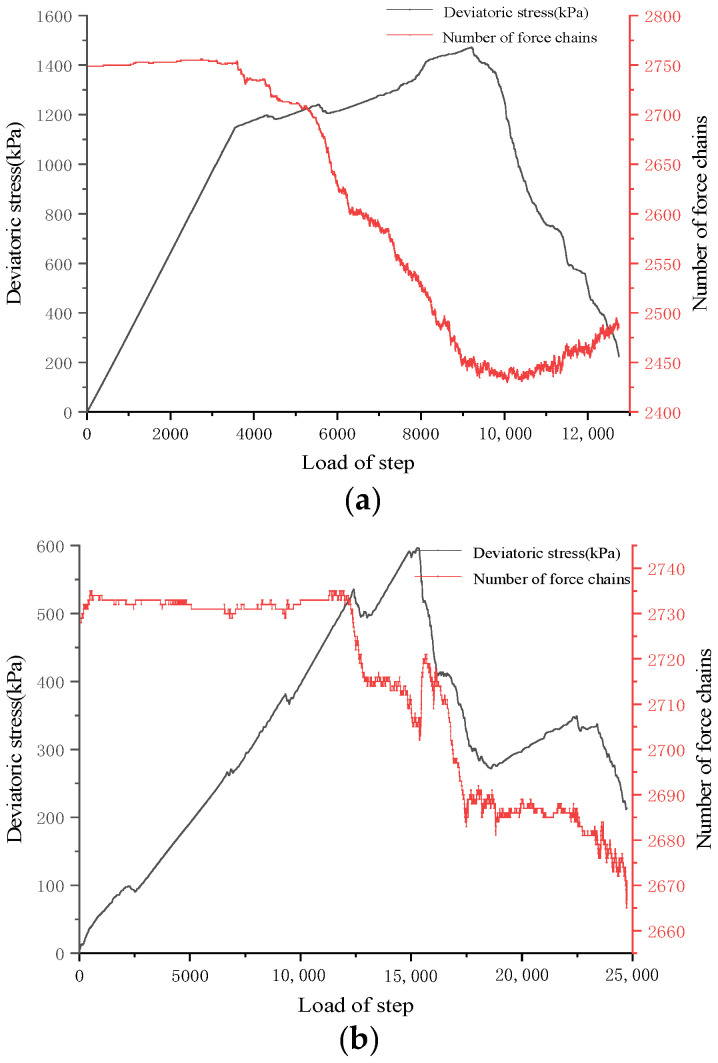
Evolution of the number of force chains in 15° and 45° rock samples under a confining pressure of 100 kPa (**a**) at a joint dip angle of 15° (**b**) and a joint dip angle of 45°.

**Figure 12:**
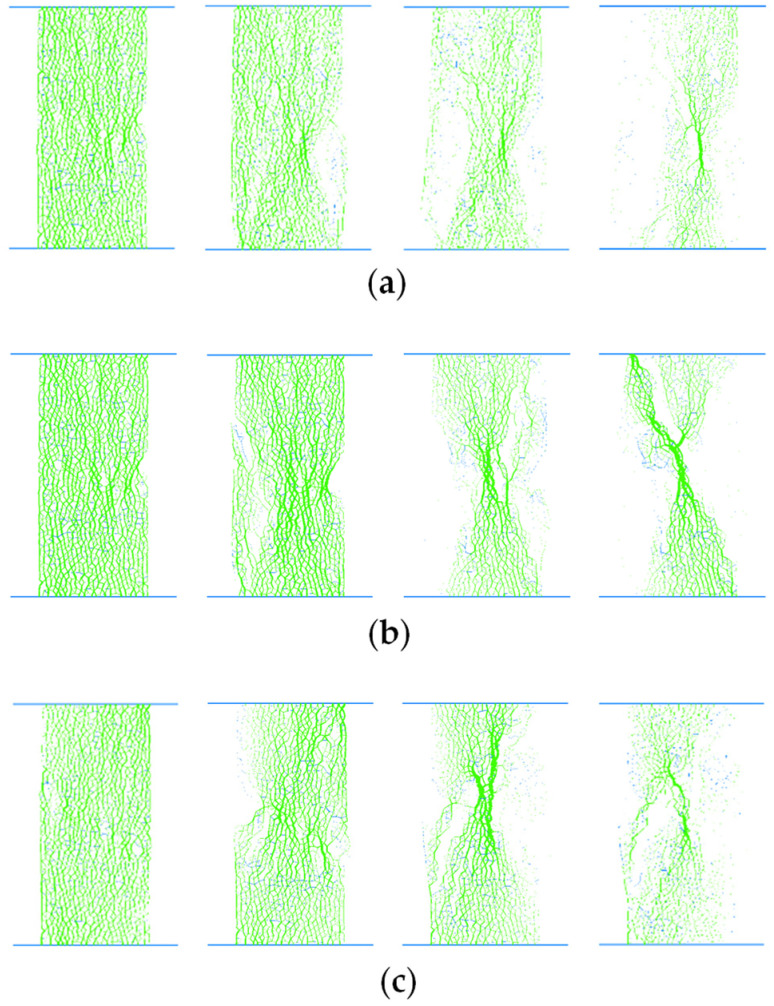

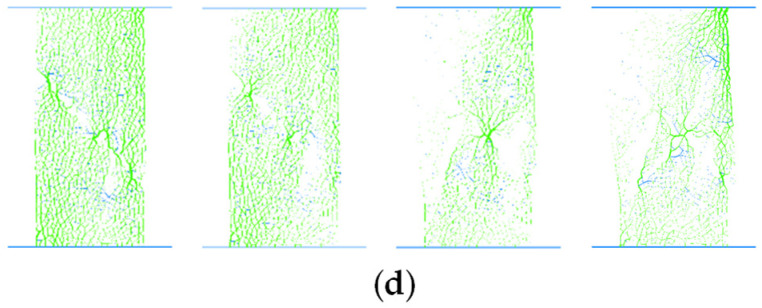
Force chains in soft rocks with varying inclinations under a confining pressure of 100 kPa. (**a**) Intact rocks (**b**) at a joint dip angle of 15°, (**c**) a joint dip angle of 30°, (**d**) and a joint dip angle of 45°.

Force chains are structures of contact forces connecting grains in a chain-like manner, representing the path of force transmission. A strong force chain is characterized by a nearly linear alignment and contact forces between grains that exceed the average force. Conversely, a weak force chain forms when contact forces are below the average. Figure 11 depicts the evolution curves of force chain numbers in soft rock with 15° and 45° joint inclinations under a confining pressure of 100 kPa. It is evident that the variation in the number of force chains for different inclinations exhibits two distinct stages. Initially, numerous force chains exist without any visible cracks, resulting in a relatively stable number of force chains; subsequently, as cracks begin to form and propagate, the number of force chains decreases continuously. Notably, temporary increases in the number of force chains may occur, which are associated with crack closure and the redistribution of forces within the rock sample. Figure 12 illustrates the changes in the distribution of force chains within rock samples with different joint dip angles under a confining pressure of 100 kPa. In this diagram, green represents pressure chains, while blue denotes tension chains. From left to right, the deviatoric stress values correspond to 90% of the peak strength in the pre-peak stage, followed by 80%, 40%, and finally 20% of the peak strength in the post-peak stage.

It is observed that main force chains are obvious in the matrix of both intact rock samples and jointed rock samples with joint angles of 15° and 30° upon failure. Conversely, the jointed rock sample with a 45° joint angle does not exhibit distinct main force chains due to the large slip along the joint plane. At the early stage of loading, the internal force chains in jointed rock samples with varying joint inclinations remain relatively intact. As the post-peak stress reaches 80% of the peak strength, cracks develop in the jointed rock samples due to the rupture of cementation bonds between particles, leading to the disruption of some force chains. When the post-peak stress decreases to 40%, significant damage is observed in all jointed rock samples, with most of the force chains being broken. In the 15° and 30° jointed rock samples, the main force chains exhibit a crosswise “X”-type distribution, whereas in the 45° rock sample, a primary force chain appears opposite the joint plane. At a post-peak stress of 20%, the jointed rock samples fail, the force chains are damaged further, and the stress transmission pathways are reduced. The peak stress values for the main stress chains in the intact rock sample and jointed rock samples with joint angles of 15°, 30°, and 45° at failure under a confining pressure of 100 kPa are approximately 6.72 MPa, 3.54 MPa, 1.73 MPa, and 1.13 MPa, respectively. These data demonstrate that, under constant confining pressure, the stress on the main stress chain of the rock sample at failure decreases as the jointing angle increases, indicating a reduced overall bearing capacity of the rock sample at failure with larger joint angles.

## 5. Conclusions

Based on the discrete element method, a series of numerical triaxial tests were conducted to investigate the macro–micro deformation and failure mechanisms of soft rocks with penetrating joints. These numerical results were compared with laboratory experiments, showing good agreement. The following conclusions were drawn.

Under constant confining pressure, the residual strength ratio of jointed rock initially increases and then decreases as the joint dip angle rises, reaching a peak at joint dip angles of 23–24 degrees. Poisson’s ratio of jointed rock increases with greater joint dip angles under constant confining pressure and also rises with increased confining pressures at the same joint dip angles. Meanwhile, the elastic modulus decreases as the joint dip angle increases under constant confining pressures but rises with higher confining pressures at constant joint dip angles. Jointed rock samples with different joint dip angles and confining pressures exhibit relatively small volumetric strains initially and then significant volume expansions before failure. Specifically, under the equivalent confining pressure, the maximum volume shrinkage is the largest in 45° jointed rock samples and the smallest in intact rock samples. Lower confining pressures and a smaller dip angle lead to more pronounced dilatancy.

As the joint dip angle increases, the degree of fragmentation at failure of soft rock decreases. Intact rock and 15° and 30° jointed soft rocks exhibit “X”-type shear failures, with strength and failure modes primarily controlled by the matrix. In contrast, 45° jointed soft rock exhibits significant sliding deformation along the joint plane initially, followed by the formation of a shear band in the matrix opposite to the joint inclination. The strength and failure mode in this case are influenced by both the matrix and the joint.

The evolution of cracks in soft rock with varying joint dip angles under different confining pressures can be categorized into three stages. The first stage is characterized by slow crack growth when the matrix remains intact. The second stage involves rapid crack propagation with a rapid increase in the number of cracks and the imminent formation of shear zones. The third stage involves through-going failure and the formation of shear failure zones. Under the same confining pressure, the number of total cracks, shear cracks, or tensile cracks decreases as the joint dip angle increases. Additionally, shear cracks are approximately twice as numerous as tensile cracks, indicating that microscopic failures are predominantly driven by shearing forces.

The evolution of force chains is closely associated with the deformation development of jointed rock. After a rapid increase in cracks, the number of force chains in jointed rock decreases. In intact soft rock and soft rock with 15° and 30° joints, a conspicuous main force chain forms within the matrix during failure. However, in 45° jointed soft rock, significant sliding along the joint plane prevents the formation of a discernible main force chain. Under the same confining pressure, stress on the main force chain during failure decreases as the joint dip angle increases, indicating that rocks with larger joint dip angles exhibit reduced bearing capacity.

## Figures and Tables

**Table 1:** PFC particle model parameters.

Particle parameters					
Minimum radius (mm)	Maximum to minimum radius ratio	Particle density (kg/m^3^)	Normal particle stiffness (MPa/m)	Stiffness ratio *k*_n_/*k*_s_	Frictional factor
1.5	1.66	2300	9	1.0	0.5
Particle bonding parameters					
Normal bond stiffness (GPa/m)	Tangential bond stiffness (GPa/m)	Bonding tensile strength (MPa)	Cohesion (MPa)	Friction angle (°)	Parallel bond radius multiplier
4	12.5	12.35	5.45	35	0.5
Parameters of smooth parallel joint models					
Normal stiffness (GPa/m)	Tangential stiffness (GPa/m)	Friction coefficient	Cohesion (MPa)		
45	24	0.5	0		

**Table 2:** Maximum residual strength ratio under different confining pressures.

Confining pressure (kPa)	100	200	300	400
Peak strength (kPa)	1097	1457	1576	1770
Residual strength (kPa)	870	1153	1291	1475
Residual strength ratio	0.7931	0.7914	0.8192	0.8333

**Table 3:** Number of cracks of jointed soft rock samples under a confining pressure of 100 kPa.

	Number of Cracks		
	Tensile Crack	Shear Crack	Total Crack
Intact	157	316	473
15°	124	250	374
30°	116	196	312
45°	38	72	110

## Data Availability

The data presented in this study are available upon request.
